# Dr. William H. Hawley

**Published:** 1914-06

**Authors:** 


Dr. William H. Hawley, Eclectic, 1850; died at his home in Penn Yan, April 26, aged 89.

